# DNA damage-induced degradation of Sp1 promotes cellular senescence

**DOI:** 10.1007/s11357-021-00456-5

**Published:** 2021-09-22

**Authors:** Michelle L. Swift, Christian Sell, Jane Azizkhan-Clifford

**Affiliations:** Department of Biochemistry and Molecular Biology, Drexel University College of Medicine, 245 N 15th Street, MS497, Philadelphia, PA 19102 USA

**Keywords:** Sp1, DNA damage, SUMOylation, Ubiquitylation, Senescence

## Abstract

Persistent DNA damage (genotoxic stress) triggers signaling cascades that drive cells into apoptosis or senescence to avoid replicating a damaged genome. Sp1 has been found to play a role in double strand break (DSB) repair, and a link between Sp1 and aging has also been established, where Sp1 protein, but not RNA, levels decrease with age. Interestingly, inhibition ATM reverses the age-related degradation of Sp1, suggesting that DNA damage signaling is involved in senescence-related degradation of Sp1. Proteasomal degradation of Sp1 in senescent cells is mediated via sumoylation, where sumoylation of Sp1 on lysine 16 is increased in senescent cells. Taking into consideration our previous findings that Sp1 is phosphorylated by ATM in response to DNA damage and that proteasomal degradation of Sp1 at DSBs is also mediated by its sumoylation and subsequent interaction with RNF4, we investigated the potential contribution of Sp1’s role as a DSB repair factor in mediating cellular senescence. We report here that Sp1 expression is decreased with a concomitant increase in senescence markers in response to DNA damage. Mutation of Sp1 at serine 101 to create an ATM phospho-null mutant, or mutation of lysine 16 to create a sumo-null mutant, prevents the sumoylation and subsequent proteasomal degradation of Sp1 and results in a decrease in senescence. Conversely, depletion of Sp1 or mutation of Sp1 to create an ATM phosphomimetic results in premature degradation of Sp1 and an increase in senescence markers. These data link a loss of genomic stability with senescence through the action of a DNA damage repair factor.

**Supplementary Information:**

The online version contains supplementary material available at 10.1007/s11357-021-00456-5.

## Introduction

Aging is defined as a series of time-related, degenerative processes beginning in adulthood that eventually end life. Aging, often referred to as organismal senescence, leads to loss of function and increases risk of diseases, such as cancer, heart disease, and diabetes [1–3]. While multifactorial in nature, aging is ultimately caused by a combination of toxic by-products of normal metabolism, such as reactive oxygen species (ROS) and imperfections in the systems that normally repair cellular damage [4–6]. DNA has long been considered as a major target of age-related cellular damage and potentially a universal cause of aging [7, 8]. Further, premature aging is a characteristic of diseases resulting from a genetic defect in DNA repair machinery, such as Cockayne syndrome, Xeroderma pigmentosa, Fanconi anemia, and Ataxia telangectasia [9–14]. The stability of the genome is maintained through a balance of repair machinery, damage tolerance, and checkpoint pathways to prevent the accumulation of DNA damage [4, 15]. Time-dependent accumulation of damage in cells and organs is associated with gradual functional decline and aging [16, 17].

Additionally, levels of ATM and its phosphorylation after ionizing radiation were found to be decreased in aged mice [18]. Moreover, both HR and NHEJ repair efficiencies have been shown to decline with age [19–25], and defects in DNA repair proteins are associated with premature aging diseases [9–14]. The molecular basis of this phenomenon is unclear and requires more active research. Here, we propose to examine the contribution of DNA damage and the systems of genome maintenance in relation to aging.

The transcription factor Sp1 has been implicated in both DNA repair and aging. The relationship between Sp1 and aging is well established. Levels of Sp1 protein, but not RNA, decrease with age [17, 26–28]. Further, Sp1 activity is decreased in aged animal tissues and senescent cells, suggesting a regulatory role for Sp1 in replicative aging [16, 17, 27]. This decrease is attributed to the accumulated effect of oxidative stress, resulting in part from deterioration in the antioxidant response in aging cells. Oxidative stress is induced by ROS, which can also generate double strand breaks (DSBs) [6, 29, 30]. Interestingly, inhibition of ataxia telangiectasia mutated kinase (ATM), the apical kinase in DSB repair, reverses the degradation of Sp1 in response to damage-induced senescence, suggesting that DNA damage signaling is involved in senescence-related degradation of Sp1 [29].

The decrease in Sp1 levels in response to DNA damage or aging is also blocked by inhibition of the proteasome [31]. Proteasomal degradation of Sp1 is mediated by the Sumo-dependent E3 ubiquitin ligase RNF4, which also localizes to sites of DSBs [32]. This degradation requires SUMOylation of Sp1 on lysine 16, which also increases with aging. Sp1^K16R^, a SUMOylation null mutant, results in increased Sp1 protein levels and cell survival [31].

We have previously reported that Sp1 is phosphorylated by ATM in response to DSBs [33]. Sp1, phosphorylated on serine 101 (Sp1^pS101^), localizes to DSBs and is necessary to facilitate DSB repair via NHEJ [34, 35]. Additionally, Sp1 is removed from break sites via damage-induced SUMOylation at lysine 16 and subsequent interaction with the SUMO-targeted E3 ubiquitin ligase, RNF4, targeting Sp1^pS101^ for proteasomal degradation [36]. Accumulation of toxic DNA damage, most notably DSBs, may be capable of driving multiple age-related phenotypes. Therefore, in the study reported here, we tested the hypothesis that degradation of Sp1^pS101^ in response to DNA damage [36] is related to the degradation of Sp1 in aged cells [29]. We further hypothesize that this damage-induced degradation of Sp1 that results in impaired NHEJ [34, 36] leads to the accumulation of DNA damage and the promotion of cellular senescence and aging. Indeed, blocking its ATM-dependent phosphorylation or its sumoylation at lysine 16 stabilizes Sp1 expression and reverses the cellular senescence phenotype [29, 31].

## Methods

### Cells

hTert-BJ1 cells (kind gift from Andrew Aplin, Thomas Jefferson University, Philadelphia, PA), which express human telomerase reverse transcriptase were cultured in high glucose Dulbecco’s modified Eagle’s medium (DMEM 10–017; Corning) containing 10% FBS (Gemini), 0.1 mg/mL penicillin, and 60 mg/mL streptomycin (Pen-Strep). Cells were incubated at 37 ˚C in a humidified atmosphere of 5% CO_2_. Human osteosarcoma cell line U2OS (ATCC) was cultured in high glucose Dulbecco’s modified Eagle’s medium (DMEM 10–017; Corning) containing 10% FBS (Gemini), 0.1 mg/mL penicillin, and 60 g/mL streptomycin (Pen-Strep). Cells were incubated at 37˚C in a humidified atmosphere of 5% CO_2_.During production of lentiviruses, HEK-293 T cells were maintained in DMEM containing 2 mM l-glutamine, 110 mg/mL sodium pyruvate, and 10% heat inactivated FBS (Gemini).

### Plasmid constructs

Viral packaging vectors, pCMV-VSV-G, pRSV-Rev, and pMDLg/pRRE, were generously donated by M. Reginato (Drexel University College of Medicine, Pennsylvania, USA). Sp1 point mutants were constructed from pLZS-Flag-Sp1. Sp1-30N^WT^, which expresses aa 1–182 of Sp1, was constructed as described previously [35]. Sp1 sgRNA constructs were constructed as described previously [34].

### Viral production

HEK293T cells were transfected with 10 μg of plasmid using GenDrill transfection reagent (BamaGen) following the manufacturer’s instructions, along with viral packaging vectors pCMV-VSV-G, pRSV-Rev, and pMDLg/pRRE (kindly provided by Mauricio Reginato, Drexel University College of Medicine, PA, USA). Virus was collected 48 h post-transfection and stored at – 80˚C.

### Antibodies and western blot analysis

Protein lysates were collected in 2 × SDS buffer (12.5 mM Tris [pH 6.8], 20% glycerol, 4% [wt/vol] SDS), and protein concentration determined by BCA assay. β-mercaptoethanol (BME) was added to lysates for a final concentration of 5%. Proteins were separated by traditional SDS-PAGE, transferred to polyvinylidene difluoride (PVDF) membrane, blocked in 5% BSA in tris-buffered saline with Tween-20 (TBST), and probed with primary antibodies overnight at 4˚C with the following antibodies: Sp1 (pAb581) [37], γH2Ax (Biolegend 613,402), α-tubulin (Cell Signaling Technology 2144), SUMO1 (Santa Cruz FL-101), RNF4 (kind gift from Ronald Hay, University of Dundee, Dundee, UK), Flag-M2 (Sigma-Aldrich F1804), p21 (Santa Cruz sc-397), lamin B1 (Santa Cruz sc-374015), phospho-Chk2 T68 (Cell Signaling Technology 2197), and Chk2 (Cell Signaling Technology 3440). Immunodetection was performed using LI-COR infrared imaging, or horse-radish peroxidase, via GeneSys G:Box F3 gel imaging system (Syngene).

### EdU and γH2Ax Immunofluorescence

BJ1 cells grown on coverslips were treated with 10 μM EdU for a total of 6 h. Cells were washed with PBS and fixed in 4% formaldehyde for 5 min at room temperature. Cells were then washed twice with PBS and permeabilized in 0.1% Triton X-100 in TBS for 10 min. Click-iT chemistry was performed by reacting EdU with the AlexaFluor Azide conjugate (ThermoFischer A10266) by incubating 100 mM Tris (pH 8.5), 1 mM CuSO_4_, 1.25 μM fluorescent azide, and 50 mM ascorbic acid (added last to the mix) in TBS. Cells were then incubated for 30 min in the dark. Cells were then washed twice with 0.1% Triton-100X in TBS and blocked for 30 min in 5% BSA at room temperature. Cells were then probed with γH2Ax antibody (Millipore Sigma 05–636) overnight at 4 ˚C. Cells were washed in PBST three times followed by the addition of secondary antibody, AlexaFluor 594—donkey anti-mouse (Invitrogen; 1:1000 in 5% BSA) for 1 h at room temperature. Cells were stained with 0.25 μg/mL DAPI (4’,6-diamidino-2-phenylindole) in PBST for 5 min and then washed three times with PBST. Slides were mounted with VectaMount mounting medium (Vector Labs). Images were obtained using EVOS FL Auto microscope (ThermoFischer).

### β-galactosidase staining

Cells were washed twice with PBS and then fixed with 4% formaldehyde for 5 min at room temperature. Cells were then washed twice with PBS and stained with X-gal (G Biosciences RC-212)(1 mg/mL of X-gal in 40 mM citric acid/Na_2_HPO_4_ [pH 6], 5 mM potassium ferrocyanide, 5 mM potassium ferricyanide, 150 mM NaCl, 2 mM MgCl_2_) for 18 h at 37 ˚C (CO_2_ free). Cells were then washed with PBS three times. Images were obtained using EVOS FL Auto microscope (ThermoFischer).

### Immunoprecipitation

The immunoprecipitation protocol was adapted from a previously described method [38]. Cells were treated with 200 µM H_2_O_2_ for 1 h. At the conclusion of treatment, cells were washed twice with PBS on ice and collected in 500 μL cold TGN buffer (50 mM Tris [pH 7.5] 150 mM NaCl, 1% Triton X-100, and protease and phosphatase inhibitors). For coimmunoprecipitation of Sp1 with SUMO1, 20 mM NEM and 4% SDS was added to the TGN buffer. Cells were disrupted 5 times with a tuberculin syringe and then sonicated twice for 30 s on/30 s off in bath a sonicator (Diagenode Bioruptor Pico). For SUMO1 coimmunoprecipitation, SDS was removed using Pierce Detergent Removal Spin Columns (Cat# 87,777). Protein was quantified using BCA assay and 1.8 to 2.5 mg of protein lysate was used for each IP. A total of 10% of the lysate was saved for input. Cells were immunoprecipitated with pre-conjugated Flag-M2 beads (Sigma A2220) or pre-conjugated SUMO1 beads (Santa Cruz sc-5308 AC) and incubated overnight at 4 ˚C. Beads were washed twice with TGN buffer. Protein was eluted with 10X SDS sample buffer (500 mM Tris [pH 6.8], 70% glycerol, and 25% [wt/vol] SDS. Precipitates were assessed by western blotting as described.

## Quantitative real time (RT) PCR

RNA was isolated from cells with Qiagen RNeasy Kit (QIAGEN). Quantitative PCR was performed with TaqMan probes (CXCL8: Hs00174103_m1; MMP3: Hs00968305_m1; CCL5: Hs99999048_m1; IL1A: Hs00174092_m1; IL6: Hs00174131_m1; GAPDH: Hs02786624_g1) using the CFX 96-real time PCR detection system (Bio Rad). Fold changes were calculated from raw data using a modified ΔΔCT method. Relative fold changes were produced by normalizing experimental fold change to the fold change of GAPDH.

### Statistical analysis

Quantification of colocalization, foci number, and colony formation was performed using ImageJ. 30 cells were counted per condition per experiment. qRT-PCR data were processed for ΔΔCt method. Data are represented as mean ± SEM and significant differences between groups were determined by two-tailed Student’s *t* test, as specified in the figure legends. *P* values are indicated by non-significant (*P* > 0.05), *(*P* < 0.05), **(*P* < 0.01), or ***(*P* < 0.001). Data without an explicit indication of statistical significance should be considered to have a *P* value greater than 0.05. All experiments were performed in triplicate, as specified in the figure legends.

## Results

### Damage-induced degradation of Sp1 promotes cellular senescence

Sp1 phosphorylated on serine 101 (Sp1^pS101^) localizes to DSBs and is necessary to facilitate DSB repair via NHEJ [34, 35]. Its role appears to be independent of transcription as expression of a truncated Sp1 mutant (aa 1–182) that lacks its DNA binding domain (Sp1-30 N), can still localize to DSBs and promote NHEJ repair [34, 35]. Additionally, levels of Sp1 protein, but not RNA, decrease with age [17, 26–28]. This decrease is attributed to the accumulated effect of oxidative stress, resulting in part from deterioration in the antioxidant response in aging cells. Sp1 is known to be phosphorylated at serine 101 (Sp1^pS101^) by the apical kinase in the DSB response, ataxia telangiectasia mutated (ATM) [33]. Inhibition of ATM reverses the degradation of Sp1 in response to damage-induced senescence, suggesting that DNA damage signaling is involved in senescence-related degradation of Sp1 [29]. Additionally, we have previously demonstrated that in response to DNA damage, Sp1^pS101^ is degraded in an S phase cell [36].

**Fig. 1:**
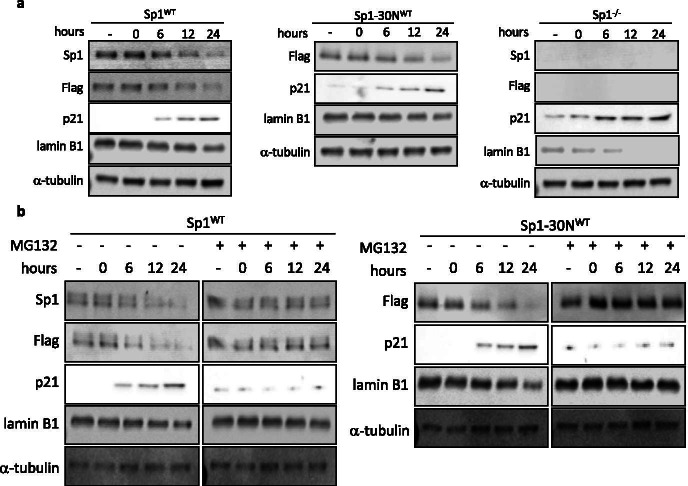
Damage-induced degradation of Sp1 results in increased p21 expression. **a**, **b** hTert-BJ1 cells were depleted of Sp1 using CRIPSR/Cas9 and transduced with lentivirus expressing Flag-tagged Sp1^WT^, Flag-tagged Sp1-30N^WT^, or empty-vector (Sp1^−/−^) (Supplemental Fig. 1a). Cells were treated with 200 μM H_2_O_2_ for 2 h, which was then replaced with fresh media for a total of 24 h. Lysates were collected at indicated time points past H_2_O_2_ removal and used for Western blot analysis of protein levels. Sp1 antibody does not detect Flag-Sp1-30 N. **b** Cells described in **a** were also treated with 10 μM MG132 for entire length of experiment

Therefore, we sought to determine if damage-induced degradation of Sp1 promotes cellular senescence. hTert-BJ1 cells depleted of Sp1 using CRISPR/Cas9 were transduced with lentivirus expressing an empty-vector (Sp1^−/−^), or lentivirus expressing Flag-tagged Sp1^WT^, or Flag-tagged Sp1-30N^WT^ under the control of its endogenous promoter (Supplemental Fig. 1a). These cells were then subjected to treatment with hydrogen peroxide (H_2_O_2_) or Adriamycin for 2 h, and lysates were then collected over a period of 24 h to assess p21 expression, a marker of cell cycle arrest, and lamin B1. Under wild-type conditions (cells expressing Sp1^WT^), we observe decreased expression of Sp1, which coincides with an increase of p21 expression and an increase in lamin B1, in response to DNA damage (Fig. 1a and Supplemental Fig. 2a). Additionally, cells expressing Sp1-30N^WT^, which lacks Sp1’s DNA binding domain and therefore cannot bind to DNA sequence specifically, displayed a decrease in Sp1 expression that also coincides with an increase in p21 expression and a decrease in lamin B1 in response to damage (Fig. 1a and Supplemental Fig. 2a). Sp1 has been shown to play a transcriptional role in cellular senescence [16, 17, 27], but since expression of Sp1-30N^WT^, which can’t bind DNA sequence specifically for transcription, does not enhance p21 expression in the absence of a DNA damaging agent suggesting that p21 expression was caused by damage-induced senescence, but independent of Sp1’s role as a transcription factor. Alternatively, in Sp1^−/−^ cells, we observe premature expression of p21 in the absence of DNA damage (Fig. 1a and Supplemental Fig. 2a). The addition of a DNA damaging agent further increases p21 expression in Sp1^−/−^ cells. These results were mimicked in U2OS cells as well (Supplemental Fig. 2b). This suggests in addition to Sp1 depletion, DNA damage is the accelerating factor required to cause the senescence phenotype. Since Sp1 is necessary for DSB repair, addition of a DNA damaging agent would cause an overwhelming amount of unrepaired DNA in Sp1^−/−^ cells, enhancing the senescence phenotype. Inhibition of the proteasome using MG132 rescued the decreased Sp1^WT^ and Sp1-30N^WT^ expression that we observed in damage-induced senescent cells, and reduced p21 expression and increased lamin B1 expression (Fig. 1b). These data suggest that damage-induced degradation of Sp1 corresponds with increased with p21 expression.

**Fig. 2:**
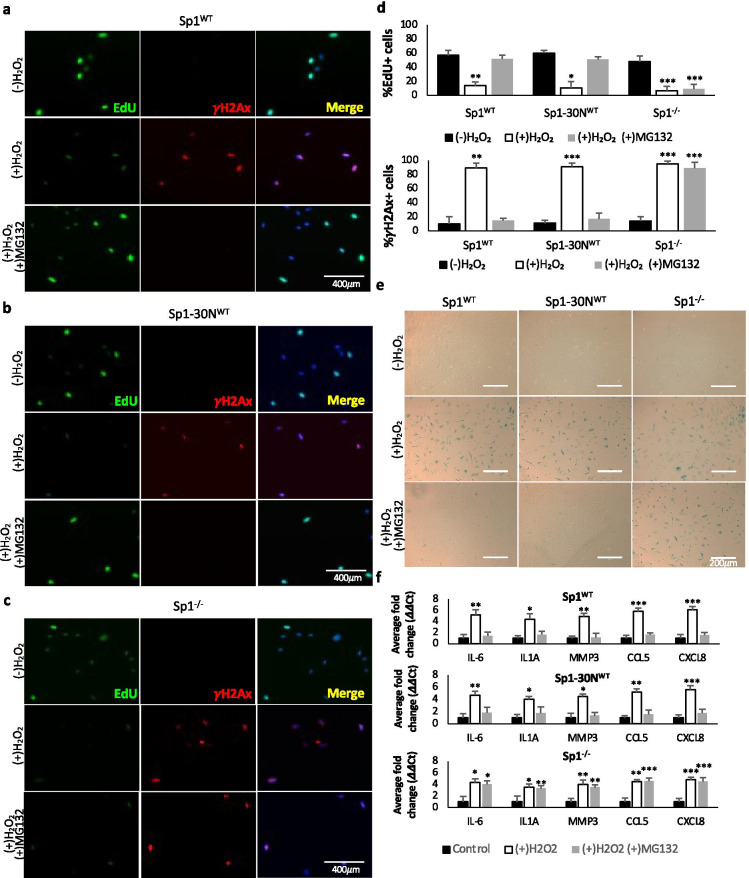
Damage-induced degradation of Sp1 promotes cellular senescence. **a**–**f** hTert-BJ1 cells were depleted of Sp1 using CRIPSR/Cas9 and transduced with lentivirus empty-vector (Sp1^−/−^), or lentivirus expressing Flag-tagged Sp1^WT^, or Flag-tagged Sp1-30N^WT^ (Supplemental Fig. 1a). Cells were treated with 200 μM H_2_O_2_ for 2 h, ± 10 μM MG132 for 24 h, then placed in fresh media for a total of 7 days. **a**–**d** 6 h prior to fixation, cells were treated with 10 μM EdU. Cells were then fixed and stained for EdU, γH2Ax, and DAPI. Scale bar represents 400 μm. **e** Cells described in a were also stained for β-galactosidase. Scale bar represents 200 μm. **f** RNA was collected from cells described above. Quantitative RT-PCR was used to analyze samples with verified primers for SASP markers. GAPDH was used as a reference gene. Data were processed for ΔΔCt method. Data represent means and SEM from 3 independent experiments assessed in triplicate. Significant differences between groups were determined by two-tailed Student’s *t* test. *, **, or *** indicate *p* values < 0.05, 0.01 or 0.001, respectively. No * indicates *p* value > 0.05

To determine if damaged-induced degradation of Sp1 promotes cellular senescence, cells depleted of endogenous Sp1 using CRIPSR/Cas9 were transduced with lentivirus expressing an empty-vector (Sp1^−/−^), or lentivirus expressing Flag-tagged Sp1^WT^, or Flag-tagged Sp1-30N^WT^. Cells were treated with H_2_O_2_ for 2 h, and EdU incorporation, γH2Ax foci, and β-galactosidase were evaluated 7 days after treatment. Cells undergoing senescence will block cell cycle progression and therefore will not cycle through S phase as evidenced by lack of EdU incorporation. Additionally, senescent cells display increased γH2Ax foci formation and increased β-galactosidase, a biomarker of cellular senescence. Consistent with p21 expression, cells expressing Sp1^WT^ or Sp1-30N^WT^ and treated with H_2_O_2_ display decreased incorporation of EdU and increased γH2Ax foci formation, compared to their non-treated controls (Fig. 2a, b, d). Addition of MG132 to cells expressing Sp1^WT^ or Sp1-30N^WT^ to inhibit damage-induced degradation of Sp1 rescued cell cycle progression and γH2Ax foci formation (Fig. 2a, b, d). The ability of MG132 to rescue the cellular senescence phenotype in cells expressing Sp1-30N^WT^ suggests that the damage-induced senescence phenotype that we are observing is not caused by defects in Sp1 transcription. Additionally, cells depleted of Sp1 (Sp1^−/−^) and treated with H_2_O_2_ exhibited decreased incorporation of Edu and an increase in γH2Ax foci formation (Fig. 2c, d). Furthermore, cells expressing Sp1^WT^ or Sp1-30N^WT^, or cells depleted of Sp1 (Sp1^−/−^) showed increased β-galactosidase staining, which could also be rescued in cells expressing Sp1^WT^ or Sp1-30N^WT^ upon the addition of MG132 (Fig. 2e).

The senescence associated secretory phenotype (SASP) is a phenotype associated with senescent cells where cells secrete high levels of inflammatory cytokines, immune modulators, growth factors, and proteases [39, 40]. We evaluated the SASP phenotype in cells expressing Sp1^WT^ or Sp1-30N^WT^, or Sp1^−/−^ cells. Cells were treated for 2 h with hydrogen peroxide, then allowed to grow for 7 days. Quantitative real time-PCR (qRT-PCR) revealed that in response to DNA damage, cells expressed increased levels of IL-6, IL1A, MMP3, CCL5, and CXCL8, inflammatory cytokines (IL-6, IL1A), matrix metalloproteases (MMP3), and chemokines (CCL5, CXCL8) associated with the SASP phenotype (Fig. 2f). In cells expressing Sp1^WT^ or Sp1-30N^WT^, addition of MG132 rescued this SASP phenotype. Together, these data suggest that damage-induced degradation of Sp1 promotes cellular senescence.

### Phosphorylation of Sp1 by ATM is necessary for Sp1 degradation and damage-associated senescence

**Fig. 3:**
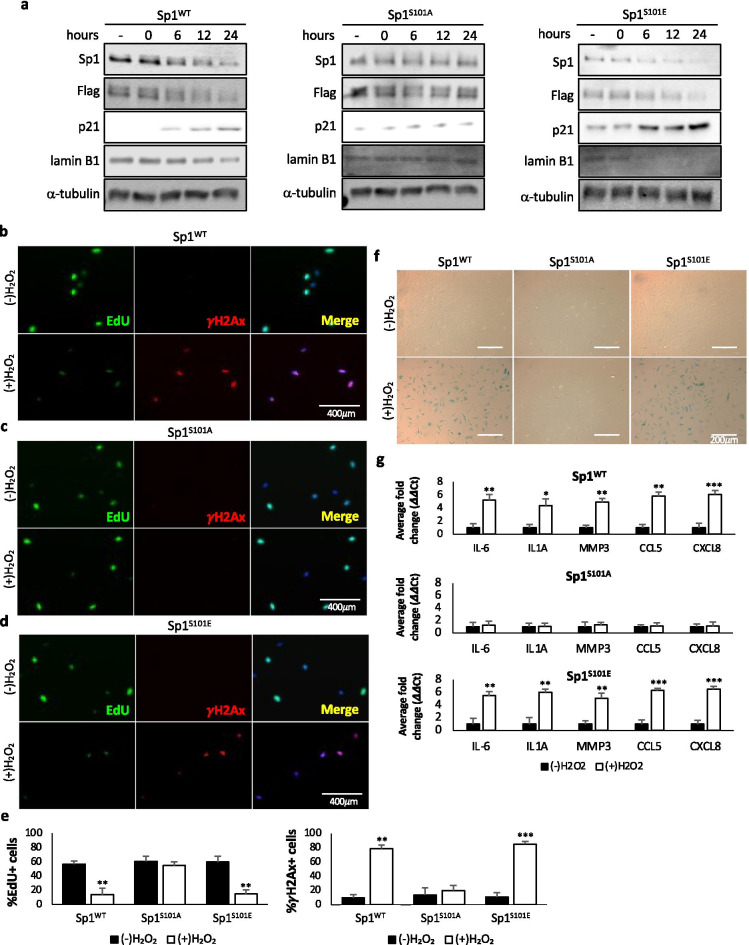
Phosphorylation of Sp1 by ATM is necessary for Sp1 degradation and damage-associated senescence. **a**–**g** hTert-BJ1 cells were depleted of Sp1 using CRIPSR/Cas9 and transduced with lentivirus expressing Flag-tagged Sp1^WT^, Sp1^S101A^, or Sp1^S101E^ (Supplemental Fig. 1a). **a** Cells were treated with 200 μM H_2_O_2_ for 2 h, then placed in fresh media for 24 h. Lysates were collected at indicated time points past H_2_O_2_ release and used for Western blot analysis of protein levels. **b**–**g** Cells were treated with 200 μM H_2_O_2_ for 2 h, then placed in fresh media for 7 days. **b**–**e** 6 h prior to fixation, cells were treated with 10 μM EdU. Cells were then fixed and stained for EdU, γH2Ax, and DAPI. Scale bar represents 400 μm. **f** Cells described above were also stained for β-galactosidase. Scale bar represents 200 μm. **g** RNA was collected from cells described above. Quantitative RT-PCR was used to analyze samples with verified primers for SASP markers. GAPDH was used as a reference gene. Data were processed using the ΔΔCt method. Data represent means and SEM from 3 independent experiments assessed in triplicate. Significant differences between groups were determined by two-tailed Student’s *t* test. *, **, or *** indicate *p* values < 0.05, 0.01, or 0.001, respectively. No * indicates *p* value > 0.05

Inhibition of ATM reverses the degradation of Sp1 in response to damage-induced senescence, suggesting that DNA damage signaling is involved in senescence-related degradation of Sp1 [29]. In response to DNA damage, Sp1 is phosphorylated by ATM at serine 101 (Sp1^pS101^). Therefore, we sought to determine the effects of damage-induced senescence on cells expressing Sp1 harboring ATM phospho-mutants at serine 101 (Supplemental Fig. 1a). Similar to cells expressing Sp1^WT^, cells expressing Sp1^101E^ displayed damage-induced degradation of Sp1, as well as increased p21 expression and decreased lamin B1 expression; but cells expressing Sp1^S101E^ displayed decreased Sp1 expression and increased p21 expression at earlier time points compared to cells expressing Sp1^WT^. In contrast, cells expressing Sp1^S101A^ did not display damage-induced degradation of Sp1 or changes in p21 or lamin B1 expression (Fig. 3a).

To further validate whether phosphorylation of Sp1 by ATM and its subsequent degradation was necessary to promote cellular senescence, we measured EdU incorporation, γH2Ax foci formation and β-galactosidase staining. Cells expressing either Sp1^WT^ or Sp1^S101E^ displayed decreased EdU incorporation and increased γH2Ax foci formation upon treatment with hydrogen peroxide (Fig. 3b, d, e). In contrast, we did not observe any changes in EdU incorporation or γH2Ax foci formation in cells expressing Sp1^S101A^ treated with hydrogen peroxide compared to non-treated (Fig. 3c, e). Additionally, in cells expressing Sp1^WT^ or Sp1^S101E^ we observed increased β-galactosidase staining in response to DNA damage (Fig. 3f).

To further validate these results, we measured markers associated with the SASP phenotype. In response to DNA damage, cells expressing Sp1^WT^ or Sp1^S101E^ expressed increased levels of IL-6, IL1A, MMP3, CCL5, and CXCL8, inflammatory cytokines, matrix metalloproteases, and chemokines, associated with the SASP phenotype, but no change in cell expressing Sp1^S101A^ (Fig. 3g). Collectively these data show that phosphorylation of Sp1 by ATM at serine 101 is necessary for damage-induced degradation of Sp1 to promote damage-associated senescence.

### Sumoylation of Sp1 is increased in response to DNA damage

**Fig. 4:**
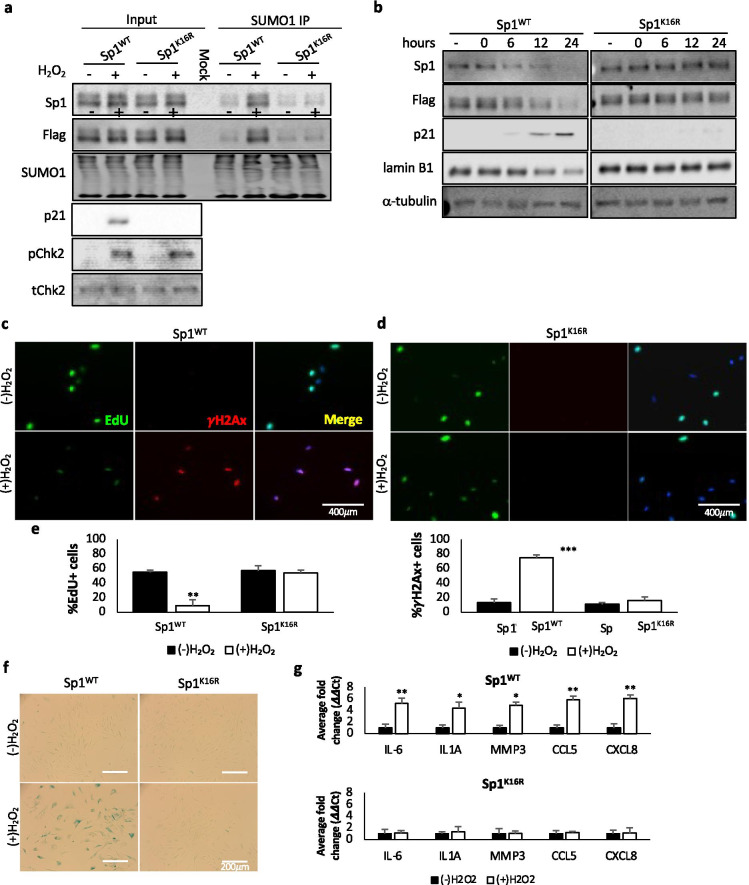
Expression of a sumo-null mutant prevents damage-induced cellular senescence. **a**–**g** hTert-BJ1 cells were depleted of Sp1 using CRIPSR/Cas9 and transduced with lentivirus expressing Flag-tagged Sp1^WT^, or Sp1^K16R^ (Supplemental Fig. 1a). **a** Following treatment with 200 μM H_2_O_2_ for 2 h, which was then replaced with fresh media for 24 h, and 10 μM MG132 for length of experiment, lysates were immunoprecipitated with SUMO1 antibody, followed by Western blotting with Flag and Sp1 to determine potential interaction with SUMO1. Phospho-Chk2 (pChk2) was used as a control to confirm DNA damage. **b** Cells were treated with 200 μM H_2_O_2_ for 2 h, which was replaced with fresh media for 24 h. Lysates were collected at indicated time points past H_2_O_2_ treatment and used for western blot analysis of protein levels. **c** − **g** Cells were treated with 200 μM H_2_O_2_ for 2 h, followed by fresh media for 7 days. **c**–**e** 6 h prior to fixation, cells were treated with 10 μM EdU. Cells were then fixed and stained for EdU, γH2Ax, and DAPI. Scale bars represent 400 μm. **f** Cells described above were also stained for β-galactosidase. Scale bars represent 200 μm. **g** RNA was collected from cells described above. Quantitative RT-PCR was used to analyze samples with verified primers for SASP markers. GAPDH was used as a reference gene. Data were processed by the ΔΔCt method. Data represent means and SEM from 3 independent experiments assessed in triplicate. Significant differences between groups were determined by two-tailed Student’s *t* test. *, **, or *** indicate *p* values < 0.05, 0.01, or 0.001, respectively. No * indicates p value > 0.05

Many DNA damage proteins are destabilized at break sites via sumoylation and subsequent recognition by a ubiquitin ligase targeting it for proteasomal degradation**.** Previously, SUMO1 was identified to covalently bind to Sp1 at lysine 16 [41, 42], resulting in its proteasomal degradation. In order to study DNA damage-induced modifications of Sp1, we examined the interaction between Sp1 and SUMO1 in response to DNA damage. hTert-BJ1 Sp1^−/−^ cells were transduced with lentivirus containing an empty vector, Flag-tagged Sp1 wild-type (Sp1^WT)^, and the Sp1 N-terminal 182 amino acid truncation, Sp1-30N^WT^, under the control of the endogenous Sp1 promoter. We also expressed Flag-tagged Sp1 sumo-null mutants, Sp1^K16R^ and Sp1-30N^K16R^, also under the control of the endogenous Sp1 promoter, as controls (Supplemental Fig. 1a). We treated cells with hydrogen peroxide for 2 h, collected lysates 24 h after treatment, and performed coimmunoprecipitation (CoIP) using a SUMO1 antibody. Western blots reveal increased interaction between SUMO1 and Sp1^WT^ and Sp1-30N^WT^ in response to DNA damage. In contrast, cells expressing Sp1^K16R^ or Sp1-30N^K16R^ we do not display increased interaction with SUMO1 in response to DNA damage (Fig. 4a and Supplemental Fig. 3a), suggesting that sumoylation of Sp1 at lysine 16 by SUMO1 is increased in response to DNA damage.

Our previous studies have shown that Sp1 is phosphorylated by ATM on serine 101 in response to damage [33]. Like other DNA damage repair proteins, phosphorylation by ATM may be a priming phosphorylation event necessary for subsequent modification by SUMO1. Therefore, we evaluated the interaction between the ATM phospho-mutants and SUMO1 in response to DNA damage. Using the ATM phospho-mutant cells (Supplemental Fig. 1a) we performed coimmunoprecipitation. In contrast to cells expressing Sp1^WT^, we observed that the phospho-null Sp1 mutant (Sp1^S101A^) does not interact with SUMO1 in the presence of DNA damage (Supplemental Fig. 3b). Additionally, compared to wild-type non-treated controls, Sp1^S101E^ displayed increased interaction with SUMO1 both in the presence and absence of damage (Supplemental Fig. 3b). Together, these data indicate that phosphorylation of the N-terminal portion of Sp1 by ATM is necessary and sufficient for its interaction with SUMO1 and subsequent sumoylation at lysine 16.

### Expression of a sumo-null mutant prevents damage-induced cellular senescence

SUMOylation of Sp1 on lysine 16 also increases with aging [31]. Sp1^K16R^, a sumo-null mutant, shows increased Sp1 protein levels and cell survival [31]. Therefore, we sought to determine the effect this sumo-null mutant had on damage-induced cellular senescence. hTert-BJ1 Sp1^−/−^ cells expressing Flag-tagged Sp1^WT^ or Sp1^K16R^ were treated with hydrogen peroxide or Adriamycin for 2 h and placed in fresh media for a total of 24 h followed by Western blotting. In contrast to cells expressing Sp1^WT^, cells expressing Sp1^K16R^ did not exhibit damaged-induced degradation of Sp1 nor any changes in p21 or lamin B1 expression (Fig. 4b and Supplemental Fig. 2a). These results were mirrored in cells expressing Sp1-30N^WT^ or Sp1-30N^K16R^ (Supplemental Fig. 3c).

We next evaluated EdU incorporation, γH2Ax foci formation, and β-galactosidase staining in cells expressing Sp1^WT^ or Sp1^K16R^ under damaged conditions. Similar to what we observed upon inhibiting the proteasome, cells expressing Sp1^K16R^ did not display defects in cell cycle progression, evidenced by EdU incorporation comparable to non-treated cells, and no increase in γH2Ax foci formation (Fig. 4c–e) or β-galactosidase staining which we observe in cells expressing Sp1^WT^ (Fig. 4f). Additionally, cells expressing Sp1^K16R^ did not display an increase in inflammatory cytokines associated with the SASP phenotype compared to cells expressing Sp1^WT^ (Fig. 4g). These data further validate that sumo-dependent degradation of Sp1 promotes cellular senescence.

### DNA damage increases Sp1’s interaction with RNF4 to facilitate Sp1 degradation and cellular senescence

**Fig. 5:**
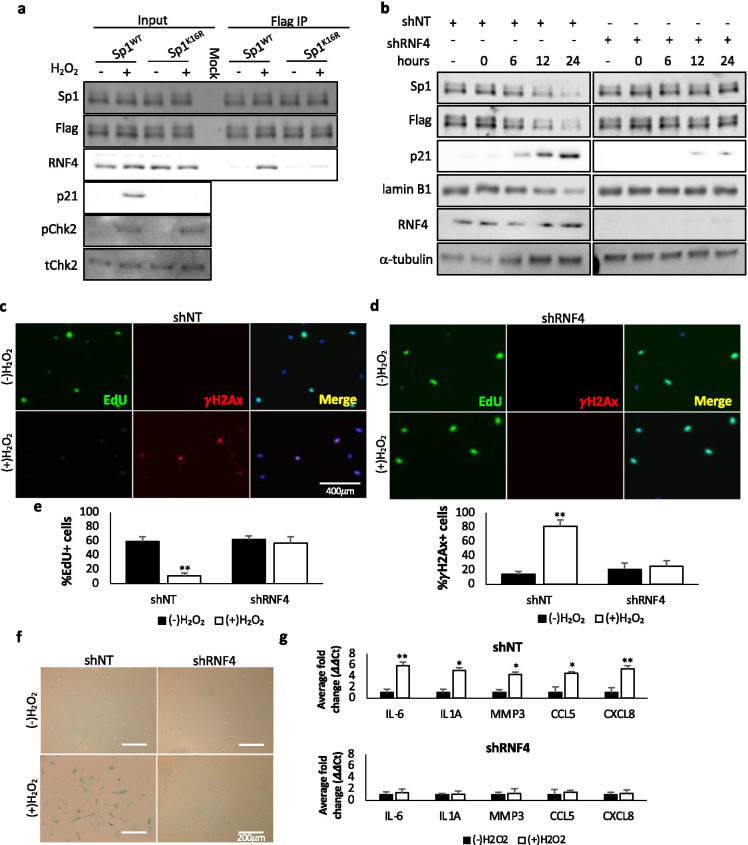
DNA damage increases Sp1’s interaction with RNF4 to facilitate Sp1 degradation and cellular senescence. **a** hTert-BJ1 cells were transduced with lentivirus expressing Flag-tagged Sp1^WT^, or Sp1^K16R^ (Supplemental Fig. 1a). Following treatment with 200 μM H_2_O_2_ for 2 h and replacement with fresh media for 24 h, and 10 μM MG132 for length of experiment, lysates were immunoprecipitated with Flag antibody, followed by western blotting with RNF4 to determine potential interaction with Sp1. Phospho-Chk2 (pChk2) was used as a control to confirm DNA damage. **b**–**e** hTert-BJ1 cells were transduced with lentivirus expressing non-targeting shRNA or shRNA against RNF4. **b** Cells were treated with 200 μM H_2_O_2_ for 2 h followed by replacement with fresh media for 24 h. Lysates were collected at indicated time points after H_2_O_2_ release and used for Western blot analysis of protein levels. **c**–**g** Cells were treated with 200 μM H_2_O_2_ for 2 h, then released into fresh media for 7 days. **c**–**e** 6 h prior to fixation, cells were treated with 10 μM EdU. Cells were then fixed and stained for EdU, γH2Ax, and DAPI. Scale bar represents 400 μm. **f** Cells described above were also stained for β-galactosidase. Scale bar represents 200 μm. **g** RNA was collected from cells described above. Quantitative RT-PCR was used to analyze samples with verified primers for SASP markers. GAPDH was used as a reference gene. Data were processed for ΔΔCt method. Data represent means and SEM from 3 independent experiments assessed in triplicate. Significant differences between groups were determined by two-tailed Student’s *t* test. *, **, or *** indicate *p* values < 0.05, 0.01, or 0.001, respectively. No * indicates *p* value > 0.05

Many DNA damage repair factor proteins are regulated by targeting them to the proteasome through SUMO-targeted ubiquitin ligases (STUbLs). These proteins target SUMO-modified proteins for ubiquitin-mediated proteolysis and are known to play a role in the DNA damage response. The STUbL RNF4 regulates various aspects of the DNA damage response (DDR) [43, 44]. Recruitment of RNF4 to damage sites is dependent on its SUMO interaction motifs (SIMs), which recognize SUMOylated substrates; the interaction between RNF4 and Sp1 is dependent on the sumoylation of Sp1 at lysine 16 (K16) [45]. We therefore sought to determine the effect of DNA damage on the interaction between Sp1 and RNF4 in cells undergoing senescence. We first confirmed that RNF4 expression did not change in senescing cells (Supplemental Fig. 4a). Coimmunoprecipitation revealed that DNA damage increased the interaction of RNF4 with both full-length Sp1^WT^ and Sp1-30N^WT^ (Fig. 5a and Supplemental Fig. 4b). To validate that sumoylation at lysine 16 is necessary for the damage-induced interaction between RNF4 and Sp1, we used Sp1^−/−^ hTert-BJ1 cells and expressed a Flag-tagged wild-type or sumo-null mutant (Sp1^K16R^) under the control of the endogenous Sp1 promoter (Supplemental Fig. 1a) and performed coimmunoprecipitation. Sp1^K16R^ did not show damage-induced interaction with RNF4 (Fig. 5a and Supplemental Fig. 4b).

To further evaluate if Sp1 phosphorylation by ATM is necessary for RNF4 interaction, we performed coimmunoprecipitation in hTert-BJ1 cells depleted of Sp1 (Sp1^−/−^) and expressing Flag-tagged Sp1^WT^, Sp1^S101A^, or Sp1^S101E^. Consistent with the observed increased interaction between Sp1^WT^ and RNF4 with damage, cells expressing the ATM site phosphomimetic, Sp1^S101E^, displayed increased interaction with RNF4 both in the presence and absence of a DNA damaging agent (Supplemental Fig. 4c). Alternatively, cells expressing Sp1^S101A^ did not display increased RNF4 interaction either in the presence or absence of a DNA damaging agent (Supplemental Fig. 4c). Together, these results suggest that ATM phosphorylation of Sp1 significantly increases its interaction with RNF4, which is necessary to target Sp1 for proteasomal degradation.

We next sought to determine the effect of RNF4 knockdown on damage-induced cellular senescence. hTert-BJ1 cells expressing either non-targeting shRNA or shRNA against RNF4 were pulse treated with hydrogen peroxide for 2 h followed by culture in fresh media for a total of 24 h. In contrast to cells expressing non-targeting shRNA, cells depleted of RNF4 did not exhibit damaged-induced degradation of Sp1 nor an increase in p21 expression (Fig. 5b), similar to what we observed in cells expressing Sp1^K16R^ (Fig. 4b). We next evaluated EdU incorporation, γH2Ax foci formation, and β-galactosidase staining in cells expressing shRNA against RNF4 under damaged conditions. Similar to what we observed in cells expressing the Sp1 sumo-null mutant (Sp1^K16R^)(Fig. 4c, d), cells depleted of RNF4 did not display defects in cell cycle progression, evidenced by EdU incorporation comparable to control cells, and no increase in γH2Ax foci formation (Fig. 5c–e). Additionally, we did not observe an increase in β-galactosidase staining or inflammatory cytokine markers associated with the SASP phenotype in cells depleted of RNF4 (Fig. 5f, g). These data further validate that damage-induced interaction between RNF4 and sumoylated Sp1 are required for the degradation of Sp1, thereby promoting cellular senescence.

## Discussion

Although it has been shown that Sp1 expression is decreased in senescent cells, the earlier studies focused on the downstream effects of Sp1 as a transcription factor. Here, we investigate a transcription-independent role of Sp1 as a DNA repair factor and how it contributes to aging. We demonstrate that ATM- and SUMO-dependent damage-induced degradation of Sp1 promotes cellular senescence.

Sp1 is recruited to DSB sites in G1 and is necessary for repair via NHEJ [34]. In an S phase cell, sumoylated Sp1 is recognized by the StUbL, RNF4, which polyubiquitylates Sp1 and targets it for the proteasome [36]. Based on these findings, we hypothesized that this damage-induced degradation was responsible for the degradation of Sp1 in senescent cells. Since senescent cells are known to have an accumulation of DNA damage, constant degradation of Sp1 would result in defective DSB repair, creating a positive feedback loop for greater accumulation of DNA damage, ultimately leading to cellular senescence.

Although we have demonstrated that damage-induced degradation of Sp1 promotes cellular senescence, the exact mechanism by which this is occurring is currently unknown. Sp1 is necessary for the recruitment of NHEJ repair factors and NHEJ [34], and it has been previously shown that NHEJ repair efficiency decreases during cellular senescence [19–25]. Therefore, continual degradation of Sp1 caused by DNA damage decreases NHEJ repair efficiency, and we propose that this is one mechanism by which Sp1 promotes cellular senescence.

Previous labs have shown that Sp1 regulates p16 expression, and that decreased Sp1 expression in senescent cells results in decreased p16 expression [46]. Interestingly, when we observe damage-induced decreases in Sp1, we see an increase in p16 expression at very early time points, suggesting that this increase is independent of So1’s transcriptional activity. There Sp1 may be involved in earlier decision points in order to help determine whether the cell undergoes senescence or apoptosis.

Additionally, 53BP1 is a mediator of cellular senescence via its interaction with p53. 53BP1’s recruitment to DSB sites is critical for its repair functions and involves auto-oligomerization and bivalent nucleosomal contacts [47]. Additionally, 53BP1 oligomerization is also crucial for its interaction with p53, which enhances genome-wide p53-dependent transcription in response to DNA damage. Defects in 53BP1-p53 interaction result in inefficient p53-dependent cell cycle checkpoint activation, ultimately leading to cellular senescence [48]. Cells depleted of Sp1 display defects in 53BP1 localization indicating that Sp1 recruits 53BP1 to DSB sites [34]. Future experiments are needed to determine if damage-induced degradation of Sp1 affects 53BP1-dependent regulation of p53 to promote cellular senescence.

Here, we provide direct evidence that the DNA damage response is tightly intertwined with cellular senescence through Sp1. Sp1 is necessary for DSB repair through its recruitment of 53BP1 for NHEJ [34]; recruitment of 53BP1 is mediated by a region of Sp1 that has no transcriptional activity. In response to damage, Sp1 is also degraded via its damage-dependent interaction with SUMO1, and recognition by the SUMO-targeted E3 ubiquitin ligase, RNF4. Increased sumoylation of Sp1 and decreased total Sp1 levels have also been associated with senescent cells [29, 31]. Importantly, we have been able to link this increase in Sp1 sumoylation at lysine 16 [31] and decrease in Sp1 expression [29] in senescent cells by demonstrating SUMO-dependent degradation of Sp1 via its interaction with RNF4. ATM-dependent phosphorylation and sumoylation of Sp1 are required for Sp1-dependent senescence, as preventing either ATM-dependent phosphorylation or sumoylation through mutation of the modified residues renders Sp1 capable of rescuing Sp1 levels and the cellular senescent phenotype. Accumulation of damage would result in overall decreased Sp1 via SUMO-dependent proteasomal degradation, resulting in defects in 53BP1 recruitment and NHEJ. Cellular senescence is associated with decreased repair efficiency as well as defects in 53BP1 recruitment via its regulation of p53, thereby providing multiple mechanisms by which Sp1’s role in the DNA damage response promotes cellular senescence.

## Supplementary Information

Below is the link to the electronic supplementary material.

Supplementary file1 (PPTX 11.3MB)

## Data Availability

The raw data and unique reagents and strains generated in this study are available from the corresponding author upon request.
