# Supplementary material for: DNA damage-induced degradation of Sp1 promotes cellular senescence
Source: GeroScience. 2021 Sep 22;44(2):683–98. doi: 10.1007/s11357-021-00456-5 (PMC9135943; doi:10.1007/s11357-021-00456-5)
Supplement: Supplementary file 1 — Supplementary file1 (PPTX 11.3MB) [file 11357_2021_456_MOESM1_ESM.pdf]

# Supplemental Figure 1

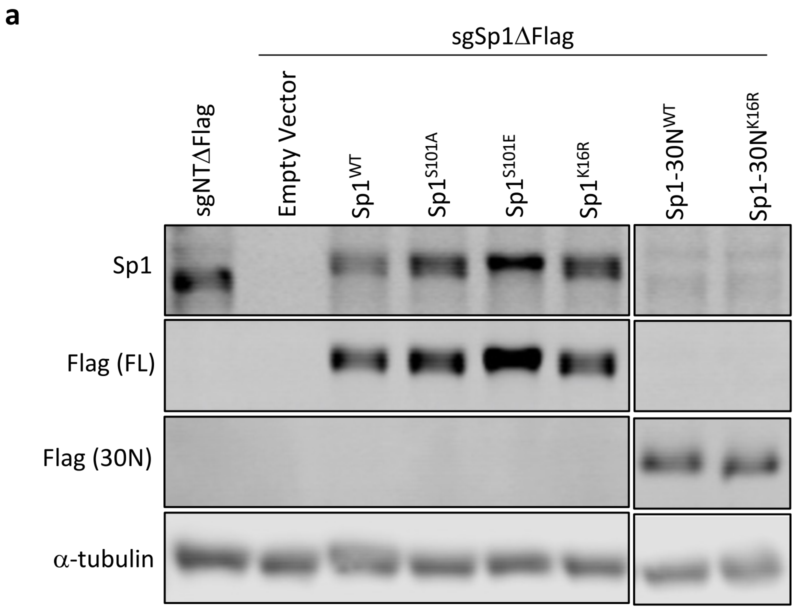


**Supplemental Figure 1: Representative Western blot of cells depleted of Sp1 and expressing Flag-tagged Sp1 and associated mutants.** (a) hTert-BJ1 cells were transduced with lentivirus containing non-targeting sgRNA or sgRNA against Sp1. Cells were then colony selected. Cells depleted of Sp1 ($Sp1^{-/-}$) were then transduced with lentivirus vector or lentivirus expressing Flag-tagged $Sp1^{WT}$ and associated mutants, and Flag-tagged $Sp1\text{-}30N^{WT}$ and associated mutants. Lysates were collected and used for Western blot analysis of protein levels. Sp1 antibody does not detect Flag-Sp1-30N.

## Supplemental Figure 2

**a**

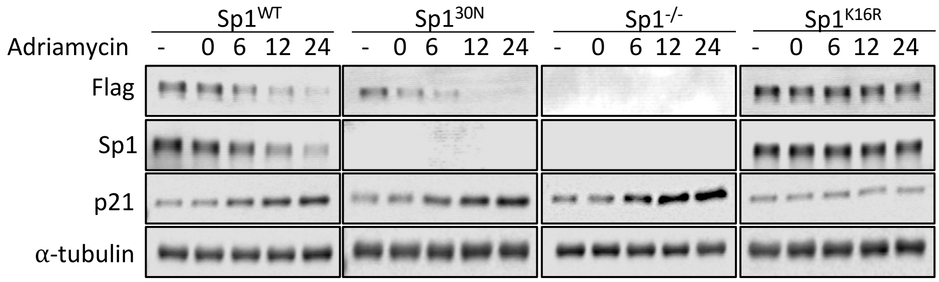


**b**

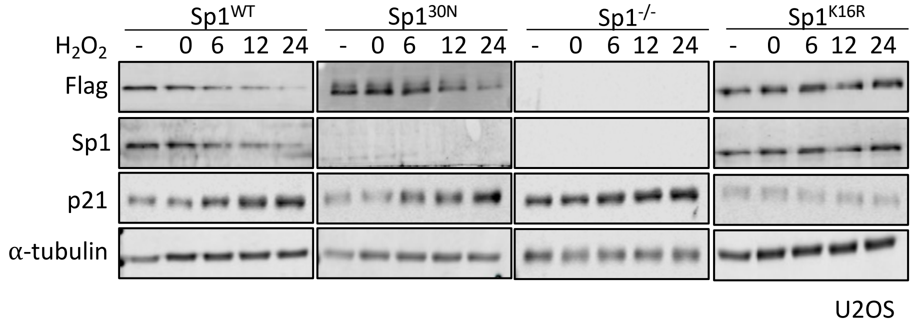


**Supplemental Figure 2: Damage-induced degradation of Sp1 results in increased p21 expression.** (a,b) hTert-BJ1 or U2OS cells were depleted of Sp1 using CRIPSR/Cas9 and transduced with lentivirus expressing Flag-tagged Sp1$^{WT}$, Flag-tagged Sp1-30N$^{WT}$ or empty-vector (Sp1$^{-/-}$) (Supplemental Figure 1a). (a) hTert-BJ1 cells were treated with 20μM Adriamycin for two hours, which was then replaced with fresh media for a total of 24 hours. Lysates were collected at indicated time points past Adiamycin removal and used for Western blot analysis of protein levels. Sp1 antibody does not detect Flag-Sp1-30N.(b) U2OS cells were treated with 200μM $H_2O_2$ for two hours, which was then replaced with fresh media for a total of 24 hours. Lysates were collected at indicated time points past $H_2O_2$ removal and used for Western blot analysis of protein levels. Sp1 antibody does not detect Flag-Sp1-30N.

# Supplemental Figure 3

a

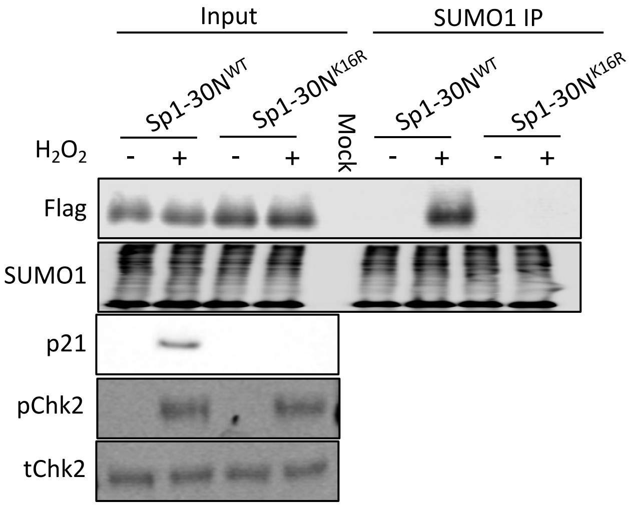


b

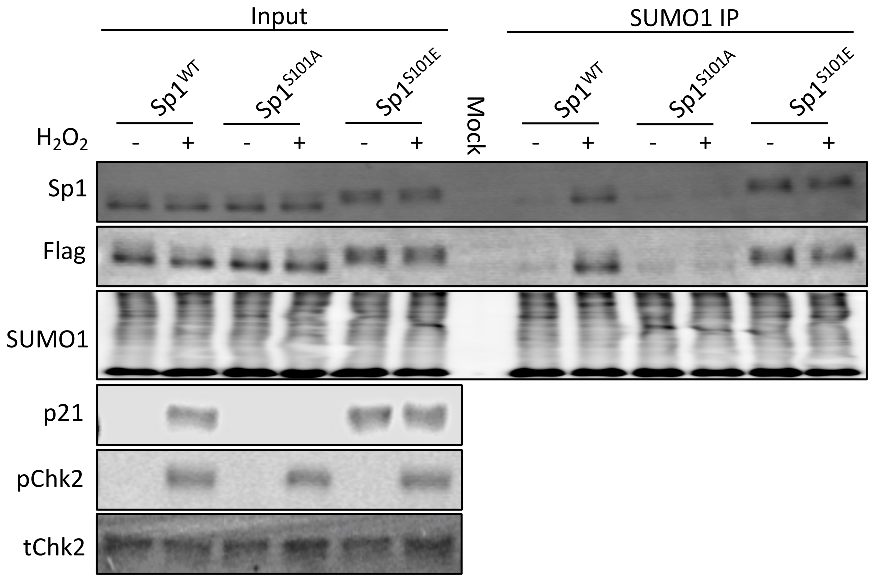


b

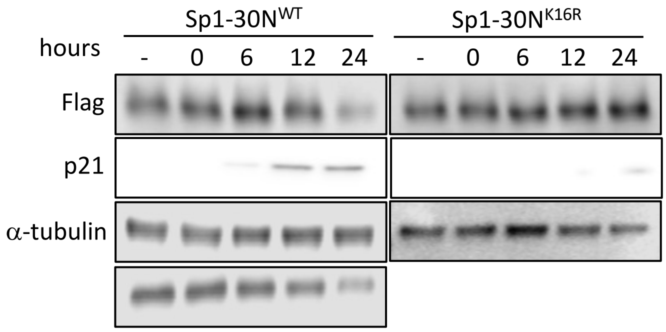

**Supplemental Figure 3: Phosphorylation of the N-terminal portion of Sp1 by ATM is necessary and sufficient for its interaction with SUMO1.** (a) hTert-BJ1 cells were depleted of Sp1 using CRIPSR/Cas9 and transduced with lentivirus expressing Flag-tagged Sp1-30N$^{WT}$, or Sp1-30N$^{K16R}$ (Supplemental Figure 1a). Following treatment with 200μM $H_2O_2$ for two hours replaced with fresh media for 24 hours, and 10μM MG132 for length of experiment, lysates were immunoprecipitated with SUMO1 antibody, followed by Western blotting with Flag and Sp1 to determine potential interaction with SUMO1 antibody. Phospho-Chk2 (pChk2) was used as a control to confirm DNA damage. Sp1 antibody does not detect Sp1-30N. (b) hTert-BJ1 cells were depleted of Sp1 using CRIPSR/Cas9 and transduced with lentivirus expressing Flag-tagged Sp1$^{WT}$, Sp1$^{S101A}$, or Sp1$^{S101E}$ (Supplemental Figure 1a). Following treatment with 200μM $H_2O_2$ for two hours, then released into fresh media for 24 hours, and 10μM MG132 for length of experiment, lysates were immunoprecipitated with SUMO1 antibody, followed by Western blotting with Flag and Sp1 to determine potential interaction with SUMO1. Phospho-Chk2 (pChk2) was used as a control to confirm DNA damage. (c) hTert-BJ1 cells were depleted of Sp1 using CRIPSR/Cas9 and transduced with lentivirus expressing Flag-tagged Sp1-30N$^{WT}$, or Sp1-30N$^{K16R}$ (Supplemental Figure 1a). Cells were treated with $H_2O_2$ for 2 hours, then released into fresh media for 24 hours. Lysates were collected at indicated time points after $H_2O_2$ treatment and used for Western blot analysis of protein levels.

## Supplemental Figure 4

a

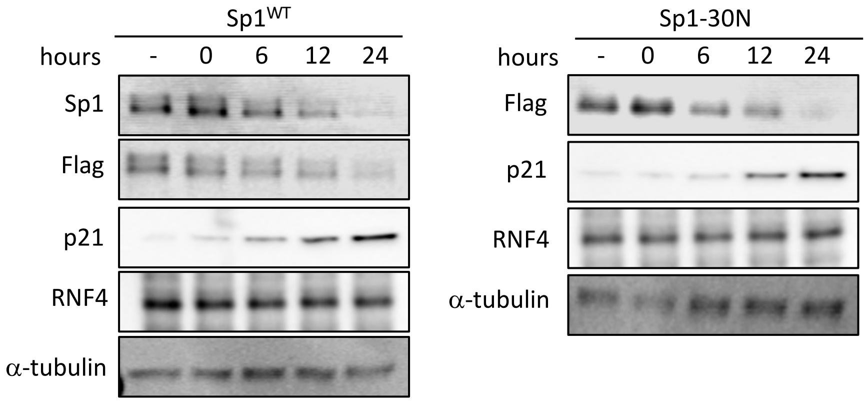


b

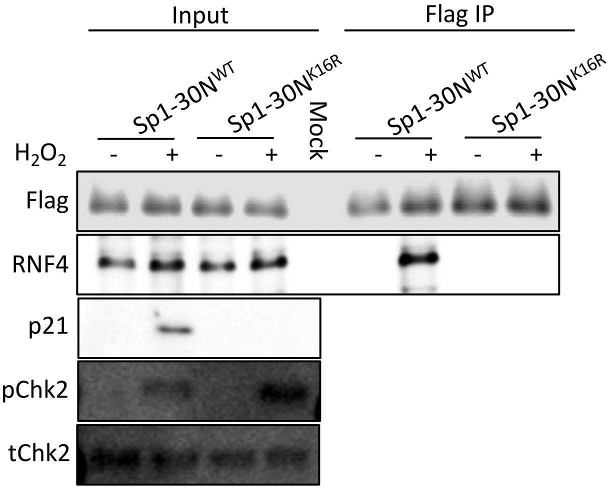


c

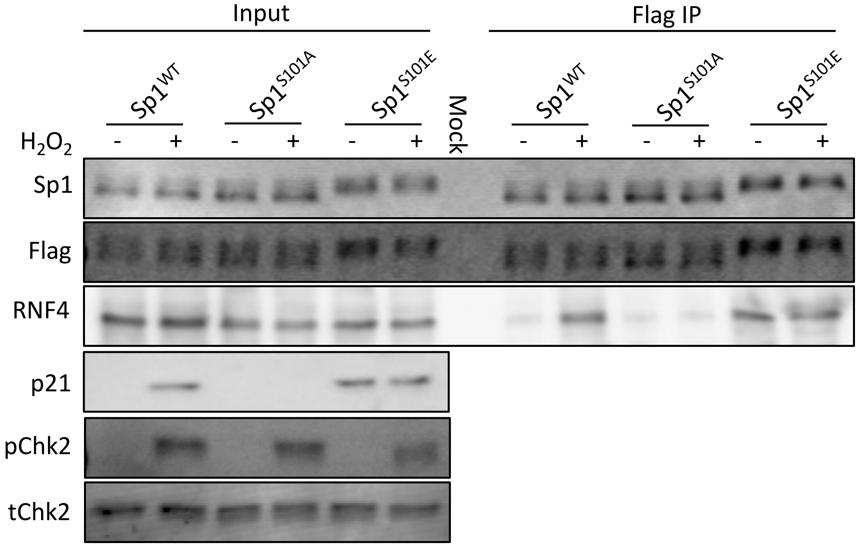

**Supplemental Figure 4: DNA damage increases the interaction between the N-terminal region of Sp1 and RNF4 to facilitate Sp1 degradation.** (a) hTert-BJ1 cells were depleted of Sp1 using CRIPSR/Cas9 and transduced with lentivirus expressing Flag-tagged $Sp1^{WT}$ or $Sp1\text{-}30N^{WT}$ (Supplemental Figure 1a). Cells were treated with $H_2O_2$ for 2 hours, followed by fresh media for 24 hours. Lysates were collected at indicated time points after $H_2O_2$ treatment and used for Western blot analysis of protein levels. (b) hTert-BJ1 cells were depleted of Sp1 using CRIPSR/Cas9 and transduced with lentivirus expressing Flag-tagged $Sp1\text{-}30N^{WT}$, or $Sp1\text{-}30N^{K16R}$ (Supplemental Figure 1a). Following treatment with 200μM $H_2O_2$ for two hours, and fresh media for 24 hours, and 10μM MG132 for length of experiment, lysates were immunoprecipitated with Flag antibody, followed by Western blotting with RNF4 to determine potential interaction with Sp1 antibody. Phospho-Chk2 (pChk2) was used as a control to confirm DNA damage. Sp1 antibody does not detect Sp1-30N. (c) hTert-BJ1 cells were depleted of Sp1 using CRIPSR/Cas9 and transduced with lentivirus expressing Flag-tagged $Sp1^{WT}$, $Sp1^{S101A}$, or $Sp1^{S101E}$ (Supplemental Figure 1a). Following treatment with 200μM $H_2O_2$ for two hours, and fresh media for 24 hours, and 10μM MG132 for length of experiment, lysates were immunoprecipitated with Flag antibody, followed by Western blotting with RNF4 to determine potential interaction with SUMO1. Phospho-Chk2 (pChk2) was used as a control to confirm DNA damage.
